# Physical activity and chronic diseases among older people in a mid-size city in China: a longitudinal investigation of bipolar effects

**DOI:** 10.1186/s12889-018-5408-7

**Published:** 2018-04-12

**Authors:** Peiling Zhou, Anne K. Hughes, Sue C. Grady, Li Fang

**Affiliations:** 1 Department of Geography, Environment, and Spatial Sciences, Michigan State University, 673 Auditorium Rd., Rm. 203, East Lansing, MI 48824 USA; 2 School of Social Work, Michigan State University, 655 Auditorium Rd., Baker Hall, East Lansing, MI 48824 USA; 3 School of Medicine, Anhui University of Science and Technology, 25 Central Dongshan Rd., Huainan, Anhui 232001 People’s Republic of China

**Keywords:** Aging, Physical activity, Chronic diseases, Longitudinal analysis, Health belief model, China

## Abstract

**Background:**

While previous studies have shown that regular physical activity can delay the onset of certain chronic diseases; less is known about the changes in physical activity practices following chronic disease diagnoses. China is experiencing a rapid aging transition, with physical activity an important routine in many older people’s lives. This study utilizes the Health Belief Model to better understand the bidirectional relationships and bipolar effects between physical activity and chronic disease burden in Huainan City, a mid-sized city in China.

**Methods:**

Longitudinal health survey data (2010–2015) from annual clinic visits for 3198 older people were obtained from a local hospital, representing 97% of the older population in three contiguous neighborhoods in Huainan City. The chronic diseases studied included obesity, hypertension, diabetes, hyperlipidemia, cardiovascular diseases, liver and biliary system diseases, and poor kidney function. Multilevel logistic regression was used to examine differences in physical activity levels across socio-demographic groups. Cox proportional hazards models were used to examine the impacts of physical activity practice levels on chronic disease onsets. Logistic regression was used to estimate the effects of chronic disease diagnosis on physical activity practice levels.

**Results:**

The prevalence of chronic diseases increased with increasing age, among men, and those with a lower education. Older people who were physically active experienced a later onset of chronic disease compared to their sedentary counterparts, particularly for obesity and diabetes. Following diagnosis of a chronic disease, physically active older people were more likely to increase their physical activity levels, while sedentary older people were less likely to initiate physical activity, demonstrating bipolar health trajectory effects.

**Conclusions:**

Health disparities among older people may widen as the sedentary experience earlier onsets of chronic diseases and worse health trajectories, compared to physically active people. Future health education communication and programmatic interventions should focus on sedentary and less healthy older populations to encourage healthy aging. These lessons from China may be applied to other countries also experiencing an increasing aging population.

## Background

The care of older people (65 years and older) with chronic disease has improved because of advanced medical technology and the promotion of healthy lifestyles. However, as mortality decreases and older people live longer, the prevalence of chronic diseases may increase, adding to the costs of a country’s health care system [1, 2]. In China in 2015, there were 14.4 million older people [3] equating to 10.5% of the total population. By mid-century, China’s older population is expected to increase to 34.1% of the total population, making China an important contributor to the global demographic transition [4]. In 2013, 49.5% of older people in China were diagnosed with at least one chronic disease [5, 6]. In 2015, the Chinese National Health and Family Planning Commission developed the National Ageing and Health Strategy and Implementation Plan to improve the health of China’s aging population through prevention programs that reduce the early onset of chronic disease, and increase well-being. The primary strategy of the Plan is to promote healthy lifestyles, through education on nutritious diets, smoking cessation, and participation in physical activity [5].

There are many studies that have investigated the impacts of physical activity (PA) on older people’s health in China [7–10]. However, only a few of these studies [7, 8] have focused on older people in mid- and small-sized cities. In these cities, residential care services are limited and there is a shortage of high-quality public health care services [11]. In addition, older people’s children may have moved out of the area, leaving their parents without family care support [12]. These two factors may be important barriers to promoting healthy lifestyle behaviors in older people in China. Studying the associations between PA and the onset of chronic disease in older people residing in mid- and small-sized cities in China can, therefore, help to target specific interventions to promote healthy aging, while also reducing the social and economic costs associated with chronic disease burden in China.

Studies have shown that regular exercise prevents chronic disease in middle-aged (54–64 years) and older people [13–15]. Regular PA has been shown to reduce the risk of cardiovascular diseases [16], hypertension and hypercholesterolemia [17], and Type 2 diabetes [17, 18] and serves as a therapy to prevent and postpone the onset of chronic disease, such as cardiovascular diseases [19], diabetes [20], and poor kidney function [21]. For older populations, PA can also reduce the risk of injuries from falls [22] and mobility disabilities [23]. Although the benefits of PA are widely cited [15, 24], sedentary lifestyles are still common among older adults, women and low-income groups worldwide [25] and older adults without a high school education in China [26]. Importantly, people may have alternative health beliefs; some people may not believe that doing PA will benefit their health so they chose to live a sedentary life in their younger years [27] attributing to a higher risk of chronic disease; some other people may only practice low-frequent and low-intensity PA for fun, therefore they are unable to achieve the full health benefits [25, 26]; some people believe that staying sedentary can reverse the disease process, thus reducing PA practice after a diagnosis of chronic disease, even though PA can attenuate or reverse the disease process to a certain extent [28]. How different health beliefs translate into positive or negative PA behaviors may in part explain health gaps during later stages of life. The bidirectional relationships between PA and chronic diseases should therefore, be considered simultaneously to understand how PA practice affects the onsets and progression of chronic diseases and how chronic diseases affect people’s PA behavior, resulting in a bipolar PA and chronic disease effect.

The purpose of this study is to investigate the bidirectional relationships between PA and different chronic disease onsets among older people in a mid-sized city in China. To study these bidirectional relationships, this study is informed by the Health Belief Model [29]. The Health Belief Model helps to distinguish attitudes and beliefs in regards to chronic disease risk and PA behaviors -e.g., older people with a chronic disease diagnosis might believe that PA will exacerbate their symptoms, worsening their disease and thereby, minimize their PA practices vs. the beliefs that PA will help to prevent or delay the onset of disease or following a diagnosis, improve their symptoms with increased PA practice. Through testing the bidirectional relationships between PA and different chronic disease onsets, future health trajectories for population groups may be inferred to inform future aging health policy and planning initiatives in China.

## Methods

### Study design

This observational study utilizes a retrospective longitudinal study design to examine the bidirectional relationships between PA and the onset of seven different chronic diseases of older residents in three neighborhoods. This study took place in three neighborhoods in Huainan City, a mid-sized city in Anhui Province, China. Huainan had a population of 1.61 million in 2014, with 16.2% of the population over 65 years of age [30]. The researchers received permission from one tier-2 public hospital in Huainan City to analyze six longitudinal years (2010–2015) of clinic data, comprising older people’s routine annual health care visits. This hospital was responsible for surveying the health of older people in their catchment area, which included three contiguous neighborhoods. There were *N* = 3198 older people in these three neighborhoods of which 97% (*N* = 3094) were included in this study. These three neighborhoods are typical of Huainan City and China (Kiu & Wu, 2006), with one old inner-city neighborhood, one *danwei* compound, and one private *xiaoqu*. This study addressed the following questions:

1. What were the prevalence of chronic diseases among older people in the three neighborhoods and how did PA vary across socio-demographic groups?

It was hypothesized that PA would vary by age, gender, education, income and the presence or absence of a chronic disease.

(2) How did the onset of a chronic disease trigger change in older people’s PA practice levels?

The hypothesis was two-fold based on the Health Belief Model –i.e., the onset of chronic disease would increase the likelihood of sedentary and/or no change in PA, or increase PA practice.

(3) Whether and to what extent was PA associated with the later onset of chronic disease?

It was hypothesized that active older adults would have a later onset of chronic disease compared to sedentary older adults.

### Data sources and population

In Huainan City, and many other cities in China, free annual medical exams begin at 55 years of age [31]. Therefore, the longitudinal health survey data included 3102 adults over 55 years (range, 55 to 99 years). Specifically, the participants include 1756 older people who completed all six years’ of clinic visits and participants who visited the clinic 2 to 5 times, which includes those with missing clinic visits (*n* = 490)--right-censored and those newly recruited (*n* = 856)—left-censored. The mean number of clinic visits for those participants who did not complete all 6 years of visits was *n* = 2.3. Since this research aims to study how the onsets of a chronic disease trigger changes in PA behaviors and how PA associated with the later onset of chronic diseases, only participants with two or more than 2 waves’ of records were considered. Eight participants were excluded from the 856 newly recruited participants due to having only one clinic visit. The final dataset comprised 3094 individual clinic visits, resulting in 13,636 observations for this study.

### Measures

Information from the health survey dataset and the coding schemes used in this study, included:

Socio-demographic (see Table 1): five year age groups (55–59, 60–64, 65–69, 70–74, 75–79, 80–84, 85–89, 90–94 and 95–99 years), gender (0 = male; 1 = female), educational level (0 = none-to-primary school; 1 = middle school; 2 = high school; and 3 = college), and self-rated income (0 = none-or-low income; 1 = high income).

**Table 1:** Description of variables and measurements of the 6-year longitudinal health survey data in Huainan

Variable Names	Code for this study	Measurement in the dataset
Socio-demographic		
Age groups	0 = 55–59, 1 = 60–64, 2 = 65–69, 3 = 70–74, 4 = 75–79, 5 = 80–84, 6 = 85–89, 7 = 90–94 8 = 95–99 years	Same
Gender	0 = male, 1 = female	Same
Educational level	0 = none-to-primary school, 1 = middle school, 2 = high school, and 3 = college	Same
Self-rated income	0 = none-or-low income, 1 = high income	Same
Physical Activity		
Levels	0 = no PA; 1 = irregular PA (not included in IPAQ categories); 2 = low PA (PA practice less than 599 MET-min/week); and 3 = moderate PA (PA practice between 600 and 3000 MET-min/week)^a^	By frequency and length^b^.

^a^ The calculation of the metabolic equivalent (MET) minute of PA per week, based on which the annual repeated PA were categorized into four levels according to the International Physical Activity Questionnaire

^b^ Frequency: 0 = no activity, 1 = irregular activity, 2 = regular activity less than three times a week. Length: 0 = no activity; 1 = less than 20 min; 2 = 20 to 60 min; 3 = 60 min or greater

Chronic Disease: To assess chronic diseases, the following data were integrated: physical data from the physical exam (height, weight and blood pressure and electrocardiogram), and peripheral venous blood samples for several chronic diseases. Seven chronic diseases were studied including, overweight and obesity (OBT), hypertension (HTN), diabetes (DBT), hyperlipidemia (HTC), cardiovascular diseases (CD), liver and biliary system disease (LBSD), and poor kidney function (PK). Overweight and obesity were calculated using the body mass index (BMI) weight/height^2^ of the individual (BMI 25.0 to 29.9 = overweight and BMI = 30 or greater = obese); hypertension was indicated by systolic blood pressure > 140 mmHg and diastolic blood pressure > 90 mmHg; diabetes was indicated by fasting plasma glucose (FPG) > 126 mg/dl; hyperlipidemia was indicated by serum total cholesterol (TC) > 240 mg/dl; cardiovascular diseases were indicated by abnormal electrocardiograph results (Yes vs. No); liver and biliary system disease was indicated by serum Alanine aminotransferase (ALT) > 40 U/L, total bilirubin (Tbil) > 1.2 mg/dl and direct bilirubin (Dbil) > 0.4 mg/dl; and poor kidney function was indicated by serum creatine (Cr) > 1.39 mg/dl and blood urea nitrogen (BUN) > 23.3 mg/dl. The normal range values provided by the hospital were used to determine if a test was normal or abnormal. These normal range values were checked against the same diseases provided in the Merck Manuals [32–37] (See detailed classification standards at [38]. For reference, the normal range values for Cr (< 1.39 mg/dl) and BUN (< 23.3 mg/dl) were slightly higher than the U.S. standards (1.2 mg/dl and 20 mg/dl, respectively) [39]. It was also recognized that some abnormal electrocardiography findings (such as Sinus arrhythmia or change on one wave) may not have indicated a cardiovascular disease, thus only those abnormal electrocardiograph findings indicated by a cardiologist on the health survey record were coded as Yes (abnormal). These disease indicators were repeatedly measured at each of the six-year time points. When a new chronic disease appeared for the first time in a given year, that year was referred to as the onset of the chronic disease. Using the normal range values provided by the hospital, the prevalence rates of the seven chronic diseases were calculated within the population, stratified by the socio-demographic groups.

Physical activity (see Table 1): To assess PA, the following data were integrated: (a) the self-reported frequency of PA per week, and (b) the self-reported length of time (in minutes) participating in PA. In the original questionnaire, PA frequency was measured as, 0 = no activity; 1 = irregular activity (randomly practicing PA); 2 = regular activity less than three times a week (stable practice of PA but infrequent); and 3 = regular daily activity (stable practice of PA and frequent); the length of activity in the survey was measured as, 0 = no activity; 1 = less than 20 min; 2 = 20 to 60 min; 3 = 60 min or greater. For this study, these two metrics were combined to calculate the metabolic equivalent (MET) minute of PA per week, based on which the annual repeated PA were categorized into four levels according to the International Physical Activity Questionnaire [40]. The PA practice levels included, 0 = no PA; 1 = irregular PA (not included in IPAQ categories); 2 = low PA (PA practice less than 599 MET-min/week); and 3 = moderate PA (PA practice between 600 and 3000 MET-min/week) [41]. In this study, MET measures –i.e., PA levels mainly represented leisure PA, excluding domestic PA (housework as PA) and occupational PA (wage-earning occupation work as PA) [9]. A PA level was assigned to each of the six-year time points, representing the overall activity level in the year prior to the clinic visit.

### Data analyses

The statistical analyses were separated into three tasks: First, prevalence rates of disease and PA levels were estimated. Binominal logistic regression was used to estimate the relationships between the prevalence rates of each of the seven disease groups and the four socio-demographic categorical variables (age group, gender, education, and income). To study the PA levels and how PA varied across socio-demographic groups, two-level multilevel binominal logistic models were estimated with annual PA levels (Level-1) nested within each individual (Level-2) [42] to predict (a) if older people were sedentary vs. practiced PA (PA level-0 vs all others), (b) if older people practiced irregular vs. regular PA (PA level-1 vs level-2 or 3, excluding level-0 observations), or (c) if older people practiced low-level vs. moderate PA (PA level-2 vs level-3, excluding level-0 or 1 observations). Since we aimed to compare sedentary (Level-0) with all other physically active groups (Level-1, Level-2 and Level-3), and to compare irregular (Level-1) with the regular PA groups (Level-2 and Level-3), three separate multilevel binominal models were estimated instead of a multinomial logistic regression model. The PA level was modeled as a fixed effect of age-group at Level-1 and of gender, education, and income at Level-2; since most individuals were retired or almost retired, there was no intrapersonal variation of income level over the six years. After deleting the participants with missing values, *n* = 3057 participants with *N* = 11,742 observations in total were used.

Second, to study the impacts of chronic disease onsets on PA, logistic models were used to estimate the change in PA, before and after the onset of a chronic disease. Since one individual could have only one onset of a certain disease and only the following year after the onset of disease would be considered for each individual, the variation within each individual was zero; models were thus reduced to a one-level logistic model. As the survey data did not include a family medical history to identify risky populations, nor a medical history with the year of a disease diagnosis prior to the beginning of the survey, only older people who had no chronic disease at baseline, and the onset of a disease in the subsequent years (*n* = 1657) were studied. The following logistic models were constructed: First, for those older people who had *no* PA (Level-0) at baseline and an onset of disease in the subsequent year, the response in model 1 was whether PA was initiated vs. sedentary in the year following the diagnosis. Second, for those older people who practiced PA (Level-1,2,3) at baseline and experienced the onset of disease in the subsequent year, the response in model 2 was whether the individual changed their PA vs. their PA remained the same, in the year following the diagnosis. Third, for those older people who changed their PA level in the year following their diagnosis in model 2, the response in model 3 was if they strengthened or lightened the intensity of their PA level, in the year following their diagnosis. For example, if the blood pressure of individual #17 exceeded the borderline value in Year 3, the onset of hypertension was allocated to Year 3, and Year 2 (baseline) and Year 1 were considered negative. These three models were estimated, controlling for age group, gender, education, and income levels.

Finally, two Cox proportional hazards models with time-varying covariates were used to examine the impacts of PA on the onsets of chronic diseases. The same initial data used in the second analysis was also used here—only older people who had no disease at baseline but reported an onset of chronic disease in the subsequent year (*n* = 1657). In both Cox proportional hazards models, the age of onset of a certain chronic disease was assigned to each individual as a survival time, since the incidence rate of chronic disease was a function of the aging process, instead of the onset time from baseline [43]. In model 1, the PA level was considered as a key independent variable. Since too few individuals (*n* = 50) conducted low-level PA, the low and moderate level activity observations went into one group -i.e. regular low to moderate PA (RPA). Finally, the three groups in model 1 were RPA, no PA (NPA), and irregular PA (IPA). In model 2, the individuals’ cumulative PA in years was calculated as the key independent variable. Since too few individuals participated in PA for over five years, Years 4, 5, and 6 were aggregated into one group. Finally, the five-year groups in model 2 were categorized as 0, 1, 2, 3, 4 or over. Gender, education and income levels were used as control variables. When a certain chronic disease was examined, all other diseases were also controlled to account for potential variation in co-morbidities. Schoenfeld residual plots were used to test the proportional hazards assumption. The statistical analyses were conducted in Stata Release 14 [44].

## Results

### Prevalence of chronic diseases by socio-demographic characteristics

In 2010, the highest chronic disease prevalence rates in the general population of older people were hypertension (51.4 per 100 older people), followed by cardiovascular diseases (38.3), overweight and obesity (31.2), diabetes (13.6), hyperlipidemia (8.9), liver and biliary system disease (7.5) and poor kidney function (4.8) (see Table 2). From the middle through older to oldest age groups, there was a statistically significant increase in prevalence of hypertension (48.2 middle age to 55.3 older to 57.7 oldest-old), cardiovascular diseases (31.9 to 43.0 to 48.9), diabetes (11.8 to 15.3 to 15.9), and poor kidney function (2.9 to 5.3 to 9.5). The prevalence of overweight/obesity and liver and biliary system disease significantly decreased after 75 years of age (31.2 to 31.9 to 26.6) and (8.2 to 7.5 to 5.7), respectively. Compared to males, females had a significantly higher prevalence of hyperlipidemia (11.3% vs. 6.6) but lower prevalence of hypertension (45.1 vs. 57.6), cardiovascular diseases (34.3 vs. 40.0), diabetes (11.5 vs. 15.4), liver and biliary system disease (5.8 vs 9.0), and poor kidney function (3.3 vs 5.8). There was not a significant difference in the prevalence of overweight and obesity by gender. After none-to-primary schooling, there was a negative linear relationship between higher education and the most highly prevalent diseases: hypertension (middle = 53.4 to high = 49.0 to college = 47.1), cardiovascular diseases (36.8 to 35.9 to 33.4) and overweight and obesity (31.5 to 30.2 to 28.3). Compared to all other educational levels, college educated older people had a significantly lower prevalence of diabetes (12.3). Education levels did not have a significant effect on the prevalence rates of hyperlipidemia, liver and biliary system disease, and poor kidney function. Finally, the prevalence of overweight and obesity was slightly higher for older people who were poor compared to higher income (31.3 vs. 30.5). There was not a significant difference in the prevalence of other chronic diseases for high income vs. none-to-low income groups, controlling for the other socio-demographic characteristics. These findings show substantial variability in chronic disease prevalence for older Chinese living in these three neighborhoods, with increasing age, male and lower education significant risk factors.

**Table 2:** Description^a^ of baseline prevalence of chronic diseases in the three neighborhoods in Huainan

		N^b^	Prevalence of Chronic Diseases %						
			OBT^c^	HTN	DBT	HTC	CD	LBSD	PK
Total		2553	31.2	51.4	13.6	8.9	38.3	7.5	4.8
Age	55–64	969	31.9	48.2	11.8	9.1	31.9	8.2	2.9
	65–74	1018	32.2	55.3***	15.3**	8.4*	43.0***	7.5	5.3***
	> = 75	566	26.6**^d^	57.7***	15.9***	8.8*	48.9***	5.7**	9.5***
Gender	Male	1385	31.2	57.6	15.4	6.6	40.0	9.0	5.8
	Female	1168	31.2	45.1***	11.6***	11.3***	34.3***	5.8***	3.3***
Education	Primary	774	33.9	55.4	14.8	9.0	42.1	7.0	5.8
	Middle	852	31.5***	53.4*	13.0	8.6	36.8*	7.5	4.8
	High	206	30.2***	49.0***	14.6	8.4	35.9**	8.0	4.5
	College	695	28.3***	47.1***	12.3*	8.7	33.4*	8.2	3.5
Income	High	414	30.5*	51.1	14.6	8.3	38.9	7.7	4.6
	Low/No	2125	31.3*	52.1	13.2	8.8	37.2	7.5	4.8

^a^ Logistic regressions were used for binominal analyses between the prevalence of chronic diseases and the four socio-demographic categorical variables

^b^*N* = 2553 residents over 55 years living in the three neighborhoods at least six months and recruited in the baseline survey

^c^OBT: overweight and obesity; HTN: hypertension; DBT: diabetes; HTC: hyperlipidemia; CD: cardiovascular diseases; LBTD: liver and biliary system diseases; PK: poor kidney function

^d^*p < 0.05, **p < 0.01, ***p < 0.001

### Physical activity and socio-demographic characteristics

Table 3 shows the estimated PA levels by socio-demographic characteristics. Importantly, as age increased, the odds that older people would practice PA was low –i.e., they were more likely to be sedentary. However, for those who practiced PA, their practice was regular (vs. irregular) and of moderate (vs. low) intensity, demonstrating a persistent and positive PA trajectory despite increasing age. In addition, the odds of PA practice for females (OR = 0.81, 95%CI:0.81–0.93, *p* < 0.01), middle (OR = 0.77, 95%CI: 0.65–0.92, *p* < 0.01) or college (OR = 0.63, 95%CI: 0.58–0.87, *p* < 0.001) educated, or older people of none-to-low income (OR = 0.71, 95%CI: 0.58–0.87, *p* < 0.001) were also low –i.e., they were more likely to be sedentary. However, for those who practiced PA, their practices were more likely to be regular, for females (OR = 1.26, 95%CI: 1.14–1.40, *p* < 0.001), college educated (OR = 1.23, 95%CI: 1.04–1.44, *p* < 0.01) and none-to-low income (OR = 1.22, 95%CI: 1.04–1.41, *p* < 0.05). There were no significant differences in PA intensity (moderate vs. low), for those practiced PA, except for college educated (OR = 0.34, 95%CI: 0.16–0.73, *p* < 0.05). These findings show that older people living in the three neighborhoods are primarily sedentary and those who practice PA, practice regularly but of varying intensity mainly by age group.

**Table 3:** Multilevel logistic regression^a^ between socio-demographic features and physical activity level among older people 2010 to 2015

		Physical Activity Level^b^					
		PA VS Sedentary		Regular VS Irregular		Moderate VS Low	
		Odds Ratio	95% CI	Odds Ratio	95% CI	Odds Ratio	95% CI
Age (years)	< 55	1		1		1	
	55–59	0.32***^d^	0.25–0.42	3.53***	2.79–4.46	2.21	0.91–5.31
	60–64	0.26***	0.20–0.35	4.64***	3.65–5.90	3.04*	1.19–7.73
	65–69	0.26***	0.19–0.35	9.25***	7.15–11.95	6.20***	2.29–16.76
	70–74	0.37***	0.27–0.52	9.34***	7.15–12.19	7.04***	2.44–20.34
	75–79	0.28***	0.20–0.40	10.46***	7.85–13.93	6.65***	2.15–20.58
	80–84	0.17***	0.11–0.25	10.95***	7.64–15.68	6.13*	1.50–25.00
	> = 85	0.13***	0.08–0.23	10.16***	6.13–16.86	18.39*	1.10–305.98
Gender	Male	1		1		1	
	Female	0.81**	0.71–0.93	1.26***	1.14–1.40	1.31	0.80–2.15
Education	No/Primary	1		1		1	
	Middle	0.77**	0.65–0.92	1.05	0.92–1.20	0.73	0.37–1.43
	High	0.91	0.67–1.21	0.97	0.79–1.19	0.50	0.18–1.33
	College	0.63***	0.51–0.78	1.23**	1.04–1.44	0.34*	0.16–0.73
Income	High	1		1		1	
	No/Low	0.71***	0.58–0.87	1.22*	1.04–1.41	0.87	0.44–1.72
N of Observations^c^		11,742		8340		5200	
n of Individuals		3057		2829		2234	

^a^In these two-level logistic regression models, the repeated PA level measure (level-1) were nested within each individual (level-2)

^b^Dependent variables are if the individual did PA (level-0 vs all others) in model 1, if the individual had regular or irregular activity (level-1 vs level-2 and 3) in model 2, or if the individual had low-level or moderate activity (level-2 vs level-3) in model 3

^c^*n* = 3057 individuals with *N* = 11,742 observations in total were used

^d^*p < 0.05, **p < 0.01, ***p < 0.001

**Fig. 1:**
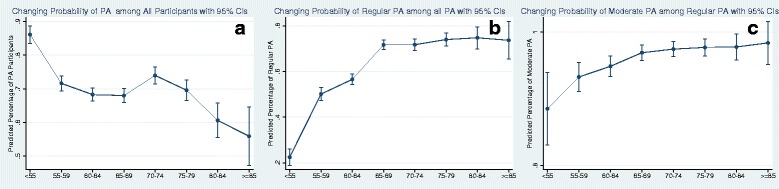
Changing odds ratios (ORs) of PA by increasing age groups. **a**: PA vs. sedentary; **b**: PA regular vs. irregular; **c**: PA moderate vs. low

When the PA intercept coefficients (data not shown) were plotted by their response variable (PA vs. sedentary, PA regular vs. irregular, and PA moderate vs. low) across all age groups, the proportion of older people practicing PA decreased (Fig. 1a) but the proportion of older people practicing regular vs. irregular and moderate vs. low PA, increased with age (Fig. 1b-c) supporting the findings above. Among those 65–79 years of age, there was a small but increasing trend in PA, but thereafter with increased age, PA decreased.

### Chronic diseases that trigger physical activity changes

Table 4 shows that for middle to older people who were sedentary at baseline, the onset of any chronic disease did not prompt them to initiate PA (OR = 0.75, 95% CI: 0.57–0.97, *p* < 0.01). In particular, the odds of initiating PA were lowest for older people diagnosed with cardiovascular disease (OR = 0.64, 95% CI: 0.41–0.95, *p* < 0.05) or liver and biliary tract disease (OR = 0.39, 95% CI: 0.16–0.94, *p* < 0.05) –i.e., were more likely to remain sedentary. However, for older people who practiced PA, the onset of any chronic disease prompted a change in PA practice (OR = 1.23, 95% CI:1.09–1.38, *p* < 0.05), in particular for hypertension (OR = 2.44, 95% CI: 1.82–3.28, *p* < 0.001), overweight and obesity (OR = 1.99, 95% CI: 1.37–2.89, *p* < 0.01), or hyperlipidemia (OR = 1.59, 95% CI: 1.24–2.02, *p* < 0.01); while the odds of changing PA practice was low for those diagnosed with diabetes (OR = 0.73, 95% CI: 0.57–0.96, *p* < 0.05), poor kidney function (OR = 0.59, 95% CI: 0.41–0.90, *p* < 0.01), or cardiovascular diseases (OR = 0.48, 95% CI: 0.39–0.58, *p* < 0.001). Of those who changed their PA practices after the onset of any chronic disease, the odds of strengthening PA practice was high (OR = 2.53, 95% CI: 2.08–3.08, *p* < 0.001), particularly for diagnoses of hypertension (OR = 3.22, 95% CI: 2.08–4.99, *p* < 0.001), overweight and obesity (OR = 2.86, 95% CI: 1.57–5.21, *p* < 0.001), hyperlipidemia (OR = 1.79, 95% CI: 1.25–2.58, *p* < 0.001), or diabetes (OR = 1.71, 95% CI: 1.04–2.84, *p* < 0.05). These results were controlling for differences in age group, gender, education and income levels. In summary, these findings show that following a disease diagnosis, sedentary older people were less likely to change their PA practices, while older people who practiced PA, also strengthened their PA practice. The PA trajectories following cardiovascular disease diagnoses were variable, with sedentary and older people who practiced PA, unlikely to change their PA practice; but those who changed their PA practice, were more likely to strengthen their practice. Importantly, for the two other highly prevalent chronic diseases—hypertension and overweight and obesity, older people were more likely to change and strengthen their PA practices.

**Table 4:** Logistic regression^a^ between the onset of chronic diseases and physical activity behaviors 2010 to 2015

	Physical Activity^b^					
	Initiate PA vs. Sedentary		Change vs. Remain		Strengthen vs. Lighten	
	Odds Ratio	95% CI	Odds Ratio	95% CI	Odds Ratio	95% CI
Any onset	0.75**^c^	0.57–0.97	1.23*	1.09–1.38	2.53***	2.08–3.08
Onset of the First	0.77	0.45–1.31	0.83	0.62–1.10	1.95**	1.17–3.24
Onset of the Second	0.94	0.61–1.44	1.10	0.88–1.38	1.89**	1.29–2.77
Onset of the Third	0.60	0.35–1.00	1.23	0.98–1.55	2.13*	1.42–3.19
Types of Diseases						
Overweight	1.10	0.39–3.21	1.99**	1.37–2.89	2.86***	1.57–5.21
Hypertension	1.33	0.60–2.96	2.44***	1.82–3.28	3.22***	2.08–4.99
Diabetes	1.03	0.5202.04	0.73*	0.57–0.96	1.71*	1.04–2.84
Hyperlipidemia	1.11	0.59–2.08	1.59**	1.24–2.02	1.79***	1.25–2.58
Cardiovascular Diseases	0.64*	0.41–0.95	0.48***	0.39–0.58	0.89	0.63–1.25
Liver& Biliary System Diseases	0.39*	0.16–0.94	0.95	0.67–1.30	1.15	0.68–1.92
Kidney Function Disorder	0.45	0.16–1.24	0.59**	0.41–0.90	0.75	0.38–1.46

^a^Three logistic regression models were used to examine the association between the change in PA and the onsets of chronic diseases controlling for age, gender, education, and income

^b^For the sedentary individuals at baseline, the response in model 1 was if he/she initiate PA in the following year after the onset of their disease; for the individuals with PA behaviors, the response in model 2 was if he/she changed or remained at their PA level in the following year; for those who did change their PA level in model 2, the response in model 3 was if they increased or decreased their PA level in the following year after the onset of a certain chronic disease

^c^*p < 0.05, **p < 0.01, ***p < 0.001

**Fig. 2:**
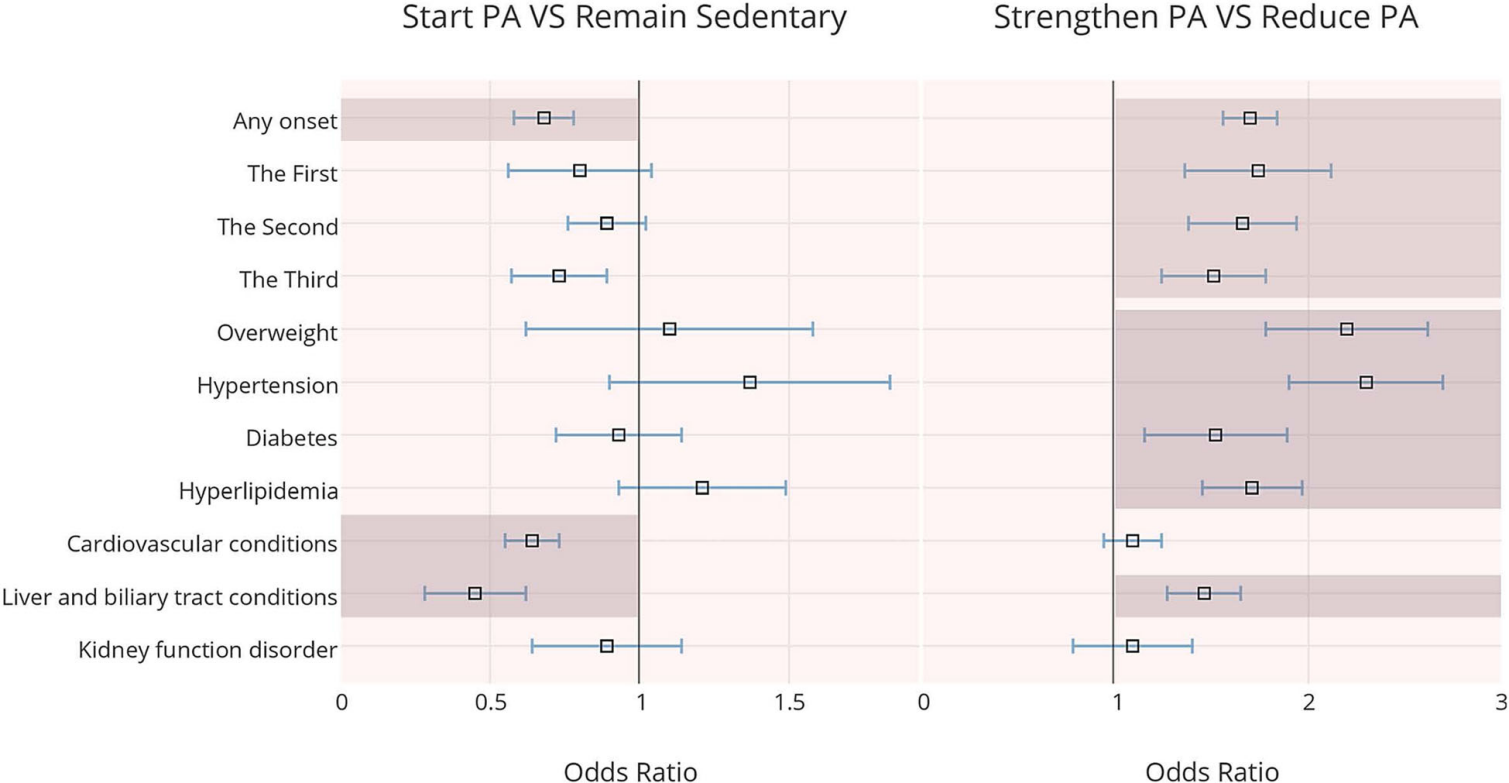
The impacts of the diagnosis of different chronic diseases upon changes in physical activity. The left figure represents the odds ratio of initiating PA vs. remaining sedentary following a diagnosis of chronic disease, for those older people who were sedentary at baseline; the right figure represents the odds ratio of strengthening physical activity versus reducing physical activity following a diagnosis of chronic disease, among those older people who were physically active at baseline

Figure 2 shows the differential impacts of a new diagnosis of chronic disease on PA change. The left figure represents the odds of initiating PA vs. remaining sedentary, for sedentary people following a new diagnosis; and the right figure represents the odds of strengthening PA vs. lightening PA among those who were previously active, following a new diagnosis.

### Physical activity and the onset of chronic disease

**Fig. 3:**
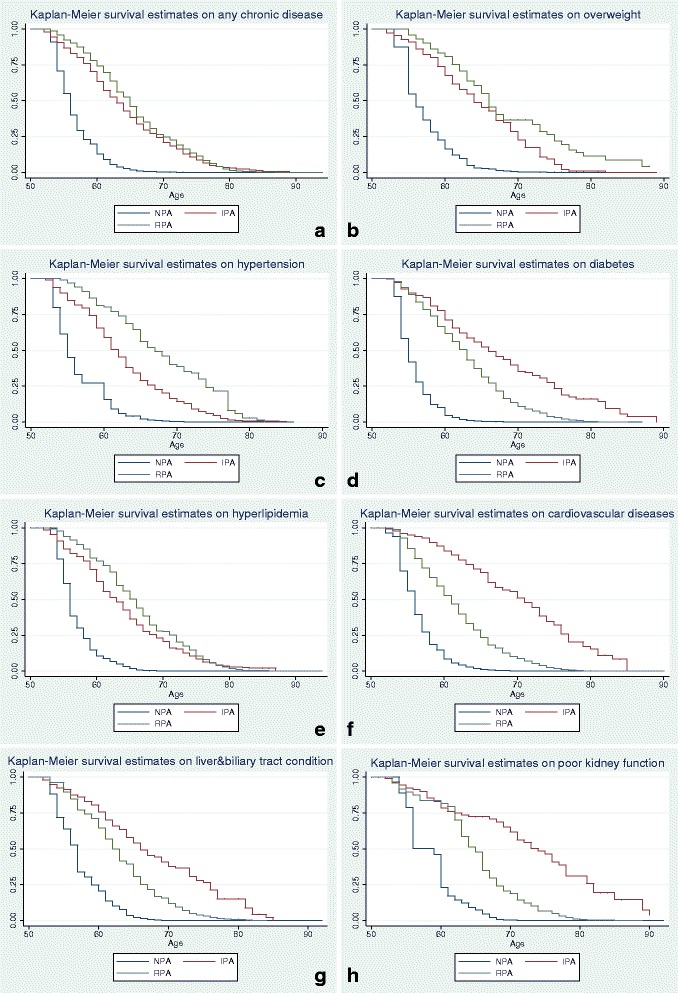
Survival estimates of different chronic diseases among people with different physical activity levels. **a**: any chronic diseases; **b**: overweight and obesity; **c**: hypertension; **d**: diabetes; **e**: hyperlipidemia; **f**: cardiovascular diseases; **g**: liver and biliary system diseases; **h**: poor kidney function

**Fig. 4:**
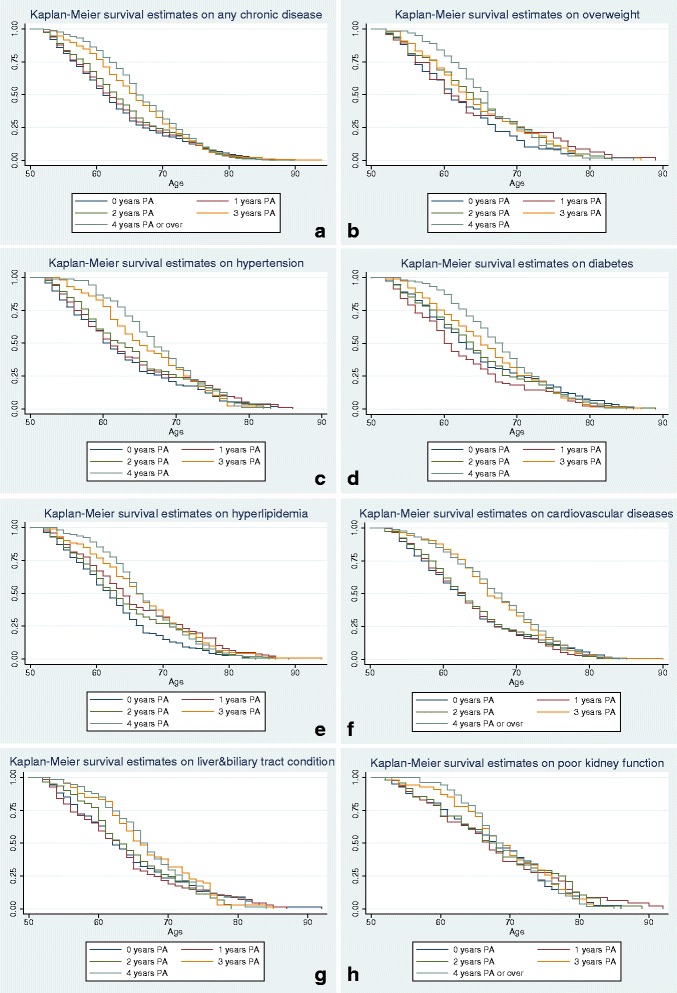
Survival estimates of onset of chronic diseases by age with different accumulative physical activity years. **a**: any chronic diseases; **b**: overweight and obesity; **c**: hypertension; **d**: diabetes; **e**: hyperlipidemia; **f**: cardiovascular diseases; **g**: liver and biliary system diseases; **h**: kidney diseases

This last analysis (Table 5) shows that both regular and irregular PA were significantly associated with later onsets of chronic disease overall (Hazard Ratio (HR) = 0.31), as well as the onsets of overweight and obesity (HR = 0.43), hypertension (HR = 0.26), hyperlipidemia (HR = 0.21), diabetes (HR = 0.17), cardiovascular diseases (HR = 0.17), poor kidney function (HR = 0.17) and liver and biliary system diseases (HR = 0.13). According to the median age (MA) of any type of onset between physically active (MA = 63) and sedentary groups (MA = 56), PA practice was significantly associated with the later onsets of chronic diseases overall by 7 years; with the later onsets of overweight and obesity and diabetes diagnoses by 8 years; with the later onsets of hypertension and hyperlipidemia diagnoses by 7 years; with the later onsets of liver and biliary system diseases and poor kidney function diagnoses by 5 years; and with the later onsets of cardiovascular disease diagnoses by 4 years (see Fig. 3). Furthermore, participating in PA over 3 year’s duration was significantly associated with a later onset of chronic diseases overall (HR = 0.21), and specifically for hyperlipidemia (HR = 0.31), cardiovascular diseases (HR = 0.16), poor kidney function (HR = 0.15), hypertension (HR = 0.14), and diabetes (HR = 0.11) (see Fig. 4). Interestingly, participating in PA for over 3 year’s duration was not significantly associated with later onsets of overweight and obesity, nor liver and biliary system diseases.

**Table 5:** ^a^Onset median age (MA) of chronic diseases and hazard ratio (HR) with different levels/years of PA

		Model 1^c^: PA level			Model 2^d^: Cumulative PA years					N of obs.	N of sub.
		NPA	IPA	RPA	0	1	2	3	4		
All Onset	MA	56	63***^e^	65***	61	62	63**	66***	66***	3414	1657
	HR^b^	1	0.31	0.4	1	0.8	0.63	0.46	0.21		
Obesity	MA	56	64***	66***	61	61	64	63	66	540	257
	HR	1	0.43	0.28	1	0.68	0.83	1.06	0.96		
Hypertension	MA	55	62***	67***	60	61*	62**	65***	67***	733	376
	HR	1	0.26	0.27	1	0.59	0.41	0.31	0.14		
Diabetes	MA	55	66***	63**	63	60*	64***	65***	68***	1069	472
	HR	1	0.17	0.6	1	0.61	0.46	0.27	0.11		
Hyperlipidemia	MA	56	63***	66***	62	64**	63	66*	66***	1143	515
	HR	1	0.21	0.28	1	0.57	0.77	0.52	0.31		
Cardiovascular diseases	MA HR	56	71***	61**	62	63**	62***	66***	68***	1965	849
		1	0.17	0.71	1	0.67	0.53	0.36	0.16		
Liver and biliary system diseases	MA	57	66***	62***	62	63	63	66	66	675	292
	HR	1	0.13	0.44	1	0.97	1.59	1.49	0.95		
Kidney function disorder	MA	59	74***	65	68	67	67	68*	68**	94	39
	HR	1	0.17	0.8	1	0.7	0.52	0.3	0.15		

^a^Two Cox proportional hazards models with time-varying covariates were used to examine the impact of PA on the onset of chronic diseases controlling for gender, education level, income level and other types of chronic diseases

^b^For each disease, the two rows report the dependent variables-onset of different diseases by onset median age (MA) and hazard ratio (HR)

^c^In model 1, the PA level was the independent variable (NPA: sedentary; IPA: irregular physical activity; RPA: Regular physical activity)

^d^In model 2 the cumulated PA years was the independent variable

^e^*p < 0.05, **p < 0.01, ***p < 0.001

## Discussion

This study is the first longitudinal study to assess the bidirectional relationships between PA and chronic diseases in an older population in a mid-sized city in China. There are three main findings from this research. First are the socio-demographic differences in the prevalence of chronic diseases and PA practices. Chronic diseases were higher among people > 65 years of age, men (excluding the diagnosis of hyperlipidemia), and those with less education. Only older people with none-to-low incomes had a higher prevalence of overweight and obesity compared to similar older people with higher incomes, but there were no significant differences in other types of chronic diseases between the two income groups. These findings suggest that early education as a prerequisite for income later in life was a primary socioeconomic driver of chronic disease burden in this older population.

There were also socio-demographic differences in PA practice, with sedentary behaviors more common with increasing age, among women, higher educated and lower income groups. However, of those women, higher educated and lower income groups who practiced PA, they were more likely to practice regularly, although of varying intensity. These findings are similar to those shown by studies at the national level in China [26, 45] and in studies worldwide [25, 46]. Reasons why older women are more sedentary than older men may be their home-life responsibilities, in particular, caring for their spouse or grandchildren. Reasons why older women who practice PA, practice regularly may have to do with their retirement age of 55 years [47], and more leisure time to conduct regular, and even daily PA. The findings on the educational and income disparities, parallel and further explain the patterns found in national studies of PA in other countries [26]. For example, this study demonstrated that low-educated older people were more likely to practice PA than middle and college-educated ones, but less likely to practice PA regularly. Plausible reasons for this finding may be that older people with less education were more likely to work in labor-intensive industries with more occupational PA so that they had good physical health for PA but less belief in the need for regular PA. High-income people were more likely to participate in PA but less likely to practice regular PA. Applicable to China, non-manual laborers usually have higher incomes than manual laborers and the former retire 5 years later than the latter. Therefore, some older people with higher incomes in this survey may not be retired, and thus having less leisure time to conduct regular PA [47], but had higher motivation for PA to keep healthy, while poor older people may have been sedentary if their basic needs were not met, and they didn’t have the physical or mental energy to practice PA. While statistical analyses in this paper are not sufficient to fully explain the educational and income disparities in PA, future studies could perhaps utilize qualitative methods to further explore these relationships.

The second main finding from this study showed that a chronic disease diagnosis was one of the most important triggers, bipolarizing PA practices between sedentary and physically active groups. In this study, a chronic disease diagnosis was associated with 32% of sedentary people remaining sedentary, indicating that sedentary older people perceived a new chronic disease diagnosis as a barrier to PA -e.g., fear that PA could exacerbate their disease symptoms and/or worsen their health. In contrast, older people who were physically active and subsequently developed a chronic disease were more likely to increase their PA level after a chronic disease diagnosis, indicating optimism that PA could reduce their symptoms of disease and/or improve their health. Enthusiasm to regulate and increase PA practice was strongest after the first chronic disease diagnosis. These findings demonstrate the complexity of attitudes and beliefs toward PA behaviors and practices for older people newly or otherwise diagnosed with a chronic disease. Understanding the personal perceptions of disease risk to one’s body following a chronic disease diagnosis, and how these perceptions differ for sedentary and active older people, may be fundamental to improving everyday PA practices in general, and following a disease diagnosis in particular, for older people ([48, 49]. Ultimately, it will be important to encourage sedentary older people to practice PA prior to a chronic disease diagnosis, to improve their health trajectory if or when a chronic disease develops.

Whether older people initiated PA or increased their PA after the onset of a chronic disease, depended on the type of diseases -i.e., in this study, people with onsets of cardiovascular and liver and biliary system diseases were less likely to be active. The attitudes and beliefs toward disease onset and progression and PA practice may be related to traditional health beliefs in China, that “people with certain diseases should relax more to keep their health” (personal observation-March 2016). Thus, education should target patients with chronic diseases, particularly those with cardiovascular and liver and biliary system diseases, so that they may still benefit from PA practices, messaging “even if the disease(s) cannot be cured”. There are also older people diagnosed with hypertension, overweight and obesity, diabetes or hyperlipidemia, who were more likely to increase (strengthen) their PA following their diagnosis. There is an important need therefore, to further educate older people about the benefits of strengthening PA after the onset of a chronic disease, particularly cardiovascular diseases [15, 19]. This education could come from family, friends, neighbors, social networks and through community activities [50, 51]—similar types of communication messaging used to encourage PA in the general, older population.

The third main finding of this study showed that older people who were physically active had significantly later onsets of chronic diseases, compared to similar older people who were sedentary. For the most prevalent chronic diseases, PA practice delayed overweight and obesity and diabetes by 8 years; hypertension by 7 years; and cardiovascular diseases by 4 years. Future PA promotion interventions should therefore, encourage sedentary older people in particular to initiate PA to delay the onset of these most highly prevalent diseases. Accurate and customized physical activity guidelines should be created and delivered to ordinary older Chinese people. Although it is not too late to initiate and regulate PA after the onset of a chronic disease, it can be more beneficial to encourage healthy people to maintain a regular PA lifestyle prior to the onset of a chronic disease [15, 16].

In summary, these findings show the bidirectional relationships between chronic disease onsets and PA, and the bipolar health trajectory effects of chronic disease onsets on PA. Under this scenario, it is likely that future health trajectories between physically active and sedentary older populations will widen as the population ages. When physically active older people were diagnosed with a chronic disease, their health trajectories appeared to be strong, as PA helped to maintain health while living with a chronic disease [15]. Comparatively, sedentary older people who experienced an onset of chronic disease, compared to their more active counterparts, were less likely to initiate PA. Although we do not know the underlying attitudes and beliefs toward disease risk and PA benefits, or physical explanations, the sedentary may have had a less healthy trajectory as their chronic disease progressed, and may therefore, need to be managed more carefully through therapeutic regimes of care.

There are some limitations of this study. The data used in this study was from a health survey in three neighborhoods in Huainan, a mid-sized city in China. Although comparisons have been made between the results of this study and other sampling studies at national level in China [26] and at global level [25], more case studies are needed in different locations, different city sizes, as well as in rural areas to provide supportive evidence for similar patterns and impacts of PA among older Chinese people. In terms of measurement, this study applied a self-reported questionnaire to measure PA. However, through the use of this measure, domestic, occupational, and other types of PA were missing from this study [9, 40]. Furthermore, the abnormal electrocardiogram results reported by the physician was the sole indicator of cardiovascular diseases, and a more robust measure could be obtained with other diagnostic criteria such as blood work and other procedures –e.g., angiogram results. Moreover, this was an observational study, so only associations between PA and socio-demographic groups and chronic disease and PA, could be found. Since this dataset excluded the highest-risk patients –i.e., those diagnosed with diseases younger than 55 years, the results may be an underestimation of the true prevalence of chronic disease risk and an overestimation of survival time, which may vary by chronic diseases. However, it is still valid to compare the relative differences in survival time within the selected disease groups, which can also demonstrate the significant positive effect of PA. Finally, geriatric syndromes were not investigated in this study. Older people’s perception of body may be experienced as more than the diagnosis of a certain chronic disease reflected in this study. As WHO’s Global Strategies on Healthy Ageing suggests [52], impairments and geriatric syndromes such as decreases in gait speed or muscle strength, which are often overlooked by health professionals, are easily perceived by older people as a problem. Reminding older people to pay attention to such syndromes could help those who are sedentary to initiate regular PA prior to the onset of chronic disease, after which it may be physically more difficult to initiate PA.

## Conclusion

In conclusion, this study highlights the prevalence of chronic disease types and PA practices by socio-demographic groups, in three neighborhoods in Huainan, a mid-sized city in China. The findings showed bidirectional relationships between chronic disease and PA, and bipolar health trajectory effects—some chronic disease diagnoses triggered PA practices, and PA practices delayed the onsets of most chronic diseases, in particular, the most highly prevalent chronic diseases—hypertension, cardiovascular diseases, overweight and obesity, and diabetes. While the National Ageing and Health Strategy and Implementation Plan aims to promote healthy lifestyles among older populations, the health beliefs that “adequate PA is not necessary to keep healthy” [27] and “PA may worsen the symptoms” [28] still commonly exist. Therefore, additional emphasis could be placed on addressing the bipolar health trajectories between physically active and sedentary older populations, identifying the differences in older people’s perceptions of PA as a risk vs. benefit to chronic disease prevention and symptom control. To improve the quality of life of older people, while reducing the economic costs of chronic disease burden, public health recommendations to encourage PA among the sedentary, and those with certain chronic disease diagnoses will be beneficial as China’s aging population increases during its rapid demographic transition. Future studies should continue to delve into the attitudes and beliefs of older people toward PA behaviors and practices. Customized physical activity guidelines could also be developed to address these attitudes and beliefs, and could be delivered to ordinary older Chinese people. These lessons from China may be applied to other countries also experiencing an increasing aging population.

## Abbreviations

ALT: Serum Alanine aminotransferase
BMI: Body mass index
BUN: Blood urea nitrogen
CD: Cardiovascular diseases
Cr: Serum creatine
Dbil: Direct bilirubin
DBT: Diabetes
FPG: Fasting plasma
HTC: Hyperlipidemia
HTN: Hypertension
IPA: Irregular physical activity
LBSD: Liver and biliary system disease
NPA: Sedentary
OBT: Overweight and obesity
PA: Physical activity
PK: Poor kidney function
RPA: Regular physical activity
Tbil: Total bilirubin
TC: Serum total cholesterol
WHO: World Health Organization
