# Correction to: Physical activity and chronic diseases among older people in a mid-size city in China: a longitudinal investigation of bipolar effects

**DOI:** 10.1186/s12889-022-12703-1

**Published:** 2022-02-28

**Authors:** Peiling Zhou, Anne K. Hughes, Sue C. Grady, Li Fang

**Affiliations:** 1 Department of Geography, Environment, and Spatial Sciences, Michigan State University, 673 Auditorium Rd., Rm. 203, East Lansing, MI 48824 USA; 2 School of Social Work, Michigan State University, 655 Auditorium Rd., Baker Hall, East Lansing, MI 48824 USA; 3 School of Medicine, Anhui University of Science and Technology, 25 Central Dongshan Rd, Huainan, Anhui 232001 People’s Republic of China; 4 Harbin Institute of Technology, Shenzhen, China

**Correction to: BMC Public Health 18, 486 (2018)**

**https://doi.org/10.1186/s12889-018-5408-7**

Following publication of the original article [1], the author identified an error in the affiliation of

- The incorrect affiliation is School of Social Work, Michigan State University, 655 Auditorium Rd., Baker Hall, East Lansing, MI 48824, USA.
- The correct affiliation is: to Harbin Institute of Technology, Shenzhen, China

The author group has been updated above and the original article [1] has been corrected.
